# Comparative evaluation of 16S rRNA primer pairs in identifying nitrifying guilds in soils under long-term organic fertilization and water management

**DOI:** 10.3389/fmicb.2024.1424795

**Published:** 2024-07-15

**Authors:** Xue Zhou, Xiaoyin Liu, Meiyu Liu, Weixuan Liu, Junzeng Xu, Yawei Li

**Affiliations:** ^1^College of Agricultural Science and Engineering, Hohai University, Nanjing, China; ^2^Jiangsu Province Engineering Research Center for Agricultural Soil-Water Efficient Utilization, Carbon Sequestration and Emission Reduction, Hohai University, Nanjing, China; ^3^The National Key Laboratory of Water Disaster Prevention, Hohai University, Nanjing, China

**Keywords:** Thaumarchaeota, primer, 16S rRNA, metagenome, paddy soil

## Abstract

Compared with 454 sequencing technology, short-read sequencing (e.g., Illumina) technology generates sequences of high accuracy, but limited length (<500 bp). Such a limitation can prove that studying a target gene using a large amplicon (>500 bp) is challenging. The ammonia monooxygenase subunit A (*amoA*) gene of ammonia-oxidizing archaea (AOA), which plays a crucial part in the nitrification process, is such a gene. By providing a full overview of the community of a functional microbial guild, 16S ribosomal ribonucleic acid (rRNA) gene sequencing could overcome this problem. However, it remains unclear how 16S rRNA primer selection influences the quantification of relative abundance and the identification of community composition of nitrifiers, especially AOA. In the present study, a comparison was made between the performance of primer pairs 338F-806R, 515F-806R, and 515F-907R to a shotgun metagenome approach. The structure of nitrifier communities subjected to different long-term organic matter amendment and water management protocols was assessed. Overall, we observed higher Chao1 richness diversity of soil total bacteria by using 515F-806R compared to 338F-806R and 515F-907R, while higher Pielou’s evenness diversity was observed by using 515F-806R and 515F-907R compared to 338F-806R. The studied primer pairs revealed different performances on the relative abundance of Thaumarchaeota, AOB, and NOB. The Thaumarchaeota 16S rRNA sequence was rarely detected using 338F-806R, while the relative abundances of Thaumarchaeota detected using 515F-806R were higher than those detected by using 515F-907R. AOB showed higher proportions in the 338F-806R and 515F-907R data, than in 515F-806R data. Different primers pairs showed significant change in relative proportion of NOB. Nonetheless, we found consistent patterns of the phylotype distribution of nitrifiers in different treatments. Nitrosopumilales (NP) and Nitrososphaerales (NS) clades were the dominant members of the AOA community in soils subject to controlled irrigation, whereas *Ca.* Nitrosotaleales (NT) and NS clades dominated the AOA community in soils subject to flooding irrigation. Nitrospira lineage II was the dominant NOB phylotype in all samples. Overall, ideal 16S rRNA primer pairs were identified for the analysis of nitrifier communities. Moreover, NP and NT clades of AOA might have distinct environmental adaptation strategies under different irrigation treatments.

## Introduction

As a pivotal step of the global biogeochemical nitrogen (N) cycle, nitrification aerobically oxidizes ammonium ( ${\mathrm{NH}}_{4}^{+}$ ) to nitrate ( ${\mathrm{NO}}_{3}^{-}$ ) through nitrite ( ${\mathrm{NO}}_{2}^{-}$ ). It influences the utilization efficiency of N fertilizer and contributes to global warming and groundwater pollution by producing nitrous oxide ( $N_{2}O$ ) and ${\mathrm{NO}}_{3}^{-}$ , respectively (Robertson and Vitousek, 2009). Traditionally, nitrification was regarded as a two-step process catalyzed by ammonia-oxidizing archaea (AOA) (Könneke et al., 2005) or bacteria (AOB) and nitrite-oxidizing bacteria (NOB). The recently discovered comammox has demonstrated that nitrification can occur within a single bacterial cell (Daims et al., 2015; Van Kessel et al., 2015). Accurately identifying the phylogeny and niche specialization of nitrifiers in complex biological and environmental samples is essential to clarifying their ecological adaptation and environmental impact.

Soils harbor a large population size and microbial diversity of prokaryotic microorganisms. A single gram of soil can harbor up to 10^10^ microbial cells (Torsvik and Øvreås, 2002) and 10^3^–10^6^ phylotypes (Bickel and Or, 2020). These mediate the biogeochemical cycling of soil mineral nutrients along with their availability. The accurate identification of microbial species and the assessment of their abundance in complex biological and environmental samples are one of the major challenges in microbiology (Knight et al., 2018). Developing high-throughput sequencing technologies has greatly increased the possibility of identifying microbial communities. Several studies have assessed microbial nitrifier communities by targeting: (i) the hypervariable region of the 16S ribosomal ribonucleic acid (rRNA) gene (e.g., Zhou et al., 2015; Cai et al., 2020), (ii) functional genes like ammonia monooxygenase subunit A (*amoA*) coding a subunit of ammonia monooxygenase (Norton, 2011), and (iii) the subunit beta of nitrite oxidoreductase (*nxrB*) gene coding the beta subunit of nitrite oxidoreductase for NOB (Pester et al., 2014). The sequencing of 16S rRNA genes could comprehensively overview the community, and quantify and characterize the relative abundance, diversity, composition and phylogenetic position of nitrifiers compared with that of functional genes.

In comparison with 454 sequencing technology, short-read sequencing (e.g., Illumina) technology generates sequences of high accuracy but limited length (<500 bp). This limitation can prove a challenge when a target gene is studied using a large amplicon (>500 bp). For example, the two sets of primers (CrenamoA23f/CrenamoA616r, amoA-AF/amoA-AR) amplify the 629-bp and 635-bp fragments of archaeal *amoA* genes, respectively, which exceeds the maximum read length of Illumina sequencing. This poses a challenge to the use of this approach in the identification of AOA microbial community. Therefore, it is necessary to evaluate whether a universal 16S rRNA gene amplicon could provide reliable information on nitrifiers, and more precisely, reflect differences in the relative abundance and composition of nitrifiers in different soils subject to various environmental factors.

The design of primers for 16S rRNA normally targets one stretch of the hypervariable regions of the 16S rRNA gene. The efficacy of various V regions in identifying microbial communities has been extensively assessed to guarantee the coverage of microbiota profiles. This is of particular importance in soils (Youssef et al., 2009; Vasileiadis et al., 2012), deep sea water (Huse et al., 2008), freshwater lakes (Zhang et al., 2018), wastewater plants (Whiteley et al., 2012) and other ecosystems. Currently, several standard protocols for environmental microbiome analysis have been developed by the Earth Microbiome Project (EMP), including the primers, amplification protocols, and conditions for the identification of prokaryotic microorganisms using paired-end 16S rRNA gene sequencing on Illumina (Thompson et al., 2017). The EMP recommends using the primer set 515F-806R for the V4 region of the 16S rRNA gene in the EMP standard pipeline (Apprill et al., 2015; Parada et al., 2016). Moreover, primer sets 338F-806R (Huse et al., 2008; Caporaso et al., 2011; Dai et al., 2022) and 515F-907R (Jing et al., 2015; Sun et al., 2015) (targeting V3-V4 and V4-V5 regions, respectively) have also been widely used for identifying and quantifying microbial communities. However, little is known about the potential bias toward nitrifiers, especially AOA, engendered by using multiple 16S rRNA gene primers targeting different V regions.

Fertilization and irrigation are important agricultural management practices that can be conducive to improving crop yields and quality. Fertilizer type (inorganic or organic fertilizer) has been recognized as a factor driving the niche separation of ammonia oxidizers. It has been demonstrated that manure amendments strongly stimulate AOA growth, while AOB is activated under inorganic fertilizer treatments (Zhou et al., 2015; Zhongjun et al., 2020), which suggests the possibility of organic carbon assimilation by AOA. Different organic substrates have been reported to exert different effects on the growth of some AOA strains (Stieglmeier et al., 2014). However, little information has been obtained on the relationship of soil AOA with manure and rice straw soil amendments, which bear relatively highly labile organic matter fractions (Yan et al., 2007) and relatively higher lignin and hemicellulose contents, respectively. Irrigation is extensively used to accommodate sustainable growth demand for grains and vegetables (Wang et al., 2007; Ibrahim et al., 2016). Different irrigation methods have different effects on soil physical properties, water content, nutrient flux, oxygen concentration, soil organic carbon, etc. (Drewry et al., 2021). These soil parameters are the factors that regulate the niche separation and activity of ammonia oxidizers (Prosser and Nicol, 2012; Qin et al., 2017). Thus, it was hypothesized that soils under different fertilization and water management protocols, which would result in distinct arrays of nitrifier communities, would provide ideal samples for evaluating primers.

In the current study, the performance of the primer pairs targeting the V3-V4, V4, and V4-V5 regions of the 16S rRNA gene was compared to analyse soil nitrifiers under different long-term organic fertilization and irrigation treatments. Moreover, although the use of the 16S rRNA gene has proved to be an efficient strategy for microbiome analysis, the PCR-based phylogenetic marker protocols are vulnerable to biases through sample preparation and sequencing errors (Langille et al., 2013). Shotgun whole genome sequencing have multiple advantages with enhanced detection of bacterial species with high accuracy and increased detection of diversity (Ranjan et al., 2016). In this study, the 16S rRNA genes obtained from the data on shotgun metagenomes without primer-induced bias were used as standards.

## Materials and methods

### Collection of soil samples

Taken from paddy fields at the Kunshan Irrigation and Drainage Experiment Station (Suzhou, Jiangsu Province, China, 31°16′ 9″ N; 120° 58′ 27″ E), soil samples were subjected to an eight-year fertilization experiment. Following the soil classification system of the World Reference Base (IUSS Working Group WRB, 2006), paddy soil was classified as hydragric anthrosol (Li et al., 2022). The experiment included four nutrient/irrigation management treatments: (i) flooding irrigation and straw return (FS); (ii) flooding irrigation and organic fertilizer (FM); (iii) controlled irrigation and straw return (CS), and (iv) controlled irrigation and organic fertilizer (CM). Controlled irrigation is a widespread local water-saving irrigation practice. The information of the upper and lower limits of paddy water conditions for water management can be observed by Liu et al. (2018). Soil with a depth of 0–10 cm was sampled in October 2022. Each treatment had three plots. Three replicates from one plot were pooled into one sample for chemical and molecular analysis. Soil samples from every plot were blended and homogenized and milled to <2 mm. The soils for physiochemical analysis were dried in the air. The soils for molecular analysis were kept at −20°C before DNA extraction.

### Measurements of soil physiochemistry

Soil water content (SWC) was gaged by oven drying at the temperature of 105°C for 24 h. Soil ${\mathrm{NH}}_{4}^{+}$ was extracted with 2 M potassium chloride (KCl) and measured according to the indophenol blue method (Ivančič and Degobbis, 1984). Soil ${\mathrm{NO}}_{3}^{-}$ was extracted with potassium sulfate (K_2_SO_4_) and measured by the salicylic acid method (Zhao and Wang, 2017). Soil pH was measured through a pH meter [Orion Star A215, Thermo Fisher Scientific, Inc., Waltham, Massachusetts (MA), the United States of America (United States)] in a 1:2 (w/v) suspension of soil to deionized water.

### DNA extraction and bacterial sequencing

DNA was extracted from 0.5 g fresh soil by using a FastDNA spin kit for soil [MP Biomedicals, Solon, Ohio (OH), United States] according to the instructions of the manufacturer. The NanoDrop ND-1000 ultraviolet–visible (UV–vis) spectrophotometer (Thermo Fisher Scientific, Waltham, MA, United States) was utilized for determining DNA concentration, followed by the storage of the extracts at −20°C for subsequent molecular analysis. Primer pairs 338F (5′-ACTCCTACGGGAGGCAGCA-3′) - 806R (5′-GGACTACHVGGGTWTCTAAT-3′), 515F (5′-GTGYCAGCMGCCGCGGTAA-3′) - 806R (5′-GGACTACNVGGGTWTCTAAT-3′) and 515F (5′-GTGCCAGCMGCCGCGG-3′) - 907R (5′-CCGTCAATTCMTTTRAGTTT-3′) were used for amplifying the V3-V4, V4, and V4-V5 regions of the 16S rRNA gene, respectively. Libraries were prepared following the protocol of the Illumina Metagenomic Sequencing Library and sequenced by Illumina NovaSeq 6000 (performed by Biomarker Technologies Corporation, Beijing, China).

### Bioinformatics analysis

Barcodes and primers were cut, and sequences were collated and clustered by use of USEARCH v11.0.667 (Edgar, 2010). A 10 bp running window was employed to trim low-quality sequence ends at the Phred quality score (Q) of 30. Paired-end reads were merged if they had greater than 10 bp overlap and no mismatches. Uchime2 was adopted to filter potential chimers (Edgar, 2016). Sequence dereplication was carried out by vsearch v2.21.1 –derep_fullength (Rognes et al., 2016). Representative ASVs (amplicon sequence variant) were extracted by using the usearch -unoise3 command. An ASV table was created by mapping the quality-filtered reads to the ASVs using the usearch -otutab command. The ASVs were taxonomically classified with the reference sequence rdp_16s_v16_sp.fa (Edgar, 2016).

### Metagenome sequencing and analysis

Roughly 30 Gb of shotgun sequence data were produced by the Hiseq 2500 platform (Illumina) in a 2 × 150 paired-end run mode. Trimmomatic v.0.38 was employed to filter and trim paired-end reads by using the following parameters: “SLIDINGWINDOW:4:20 MINLEN:50” (Bolger et al., 2014). MEGAHIT (v1.2.9) was used for assembling the filtered clean reads for each metagenome with default parameters (Li et al., 2016). Barrnap v0.9^1^ was applied to extract bacterial and archaea 16S rRNA sequences. All the extracted 16S rRNA sequences were annotated using vsearch v2.21.1 with usearch_global command (Rognes et al., 2016) based on the reference sequence rdp_16s_v16_sp.fa. The transcript per million (TPM) of 16S rRNA sequences was calculated by using bwa (v.0.7.17) (Li and Durbin, 2010) and samtools (v.1.7) (Li et al., 2009). The relative abundance of each taxon was calculated based on TPM value.

### Statistical analysis

R v3.5 (R Core Team, 2013) was used to perform statistical tests. Estimates of alpha diversity (Chao1 richness and Pielou’s evenness) were computed based on sequence normalization. The sequences were normalized to match the sample with the lowest read count (75,000 reads). Nonmetric multidimensional scaling (NMDS) was used to visualize Bray–Curtis dissimilarity matrices based on the overall ASV table and nitrifiers’ ASV subsets, respectively. Analysis of similarity (ANOSIM) was adopted to evaluate the significant differences in the community structure of bacteria, AOA, AOB, and NOB. It was considered that differences at *p* < 0.05 showed statistical significance. ANOVA based on the Turkey HSD test was conducted to test the difference in the diversity of total bacteria among different primer pairs and treatments. To compare the proportion of Thaumarchaeota, AOB and AOA in a plot, we calculated log_2_-transformed ratios of the proportion of the taxon from amplicon sequencing data (from 338F-806R, 515F-806R, or 515F-907R), as similarly used previously (Zhao et al., 2023). MEGA 11 software was used for constructing a phylogenetic tree using the neighbor-joining (NJ) method. Raw 16S rRNA gene amplicon sequencing and metagenome data were deposited to the National Center for Biotechnology Information (NCBI). Accession numbers are established in Supplementary Table S3.

## Results

### Soil chemical properties

The difference in irrigation methods significantly influenced soil SWC and pH, and soils with flooding irrigation treatment had higher SWC and lower pH than those with controlled irrigation treatment (Supplementary Table S1). Fertilizer type influenced soil total carbon (TC) and nitrogen (TN) contents. TC content was higher in the soils with organic fertilizer, while TN content was higher in the soils with straw. Consequently, the C/N ratio was higher in the soils with organic fertilizer. No significant difference was detected in NH_4_^+^ content among all treatments.

### Comparison of total bacterial communities from 16S rRNA amplicon sequences

**Figure 1:**
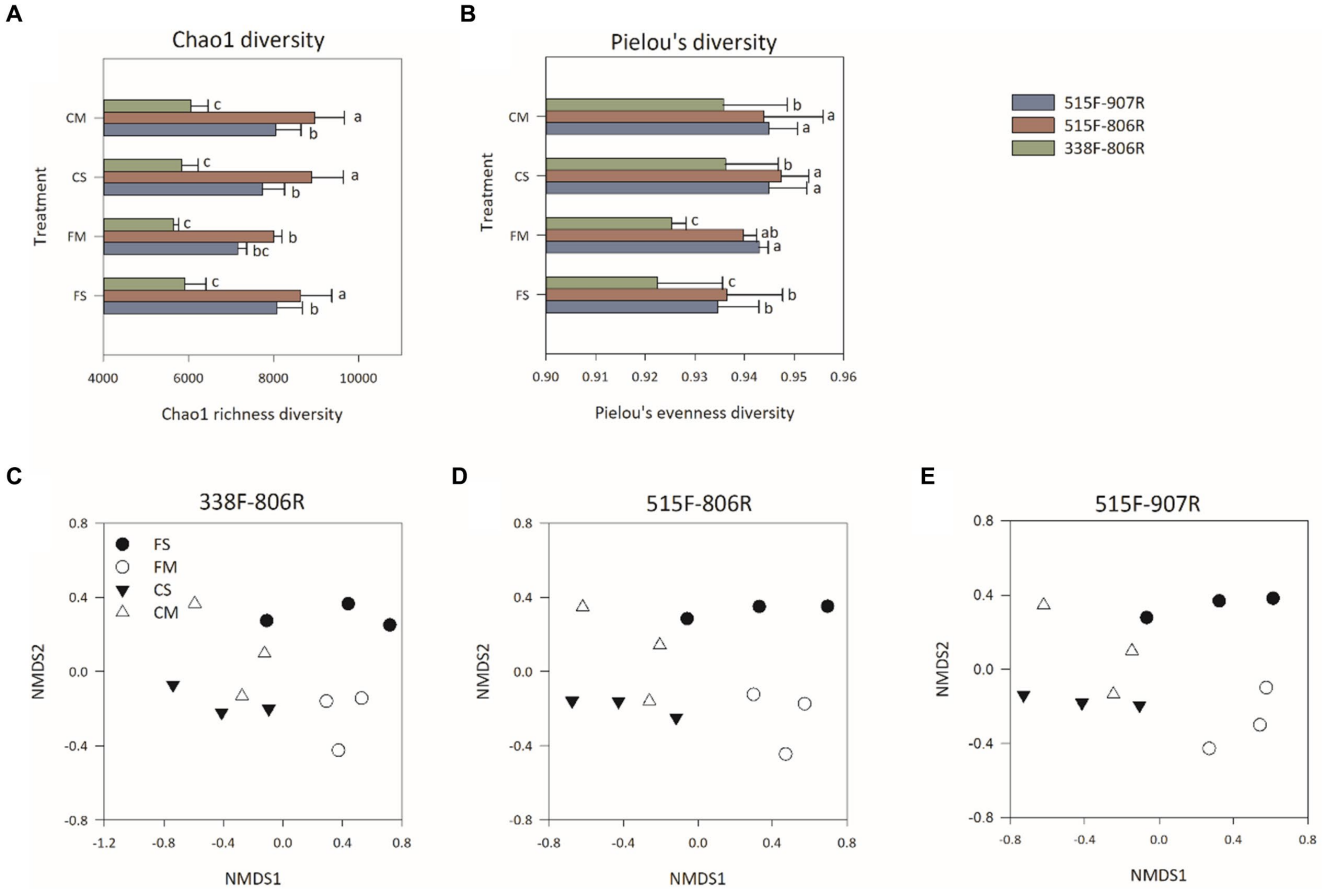
Alpha diversity [Chao1 richness diversity **(A)** and Pielou’s evenness diversity **(B)**] and Non-metric multidimensional scaling (NMDS) analysis of bacterial community in different treatments by using 338F-806R **(C)**, 515F-806R **(D)**, and 515F-907R **(E)**, respectively. The designation FS and FM indicate flooding irrigation and straw returning management and flooding irrigation and organic fertilizer management, respectively. The designation CS and CM indicate controlled irrigation and straw returning management and controlled irrigation and organic fertilizer management, respectively. The error bars represent the standard deviation for each treatment. Different letters above bars indicate statistically significant differences (*p* < 0.05) by ANOVA analysis.

Overall, 79,710 ± 211, 80,039 ± 232, and 80,021 ± 151 reads per sample were retrieved from primer sets 308F-806R, 515F-806R, and 515F-907R, respectively, after trimming and chimeral removal. The use of 515F-806R and 515F-907R resulted in significantly higher Chao1 richness and Pielou’s evenness compared to that of 338F-806R (*p* < 0.05) (Figures 1A,B). The non-metric multidimensional scaling (NMDS) analysis (Figures 1C–E) and the ANOSIM results showed that no significant difference in microbial community structure among different treatments was observed (*p* < 0.05) (Supplementary Table S4).

### Comparison of the relative abundance of nitrifiers from 16S rRNA amplicon sequences

**Figure 2:**
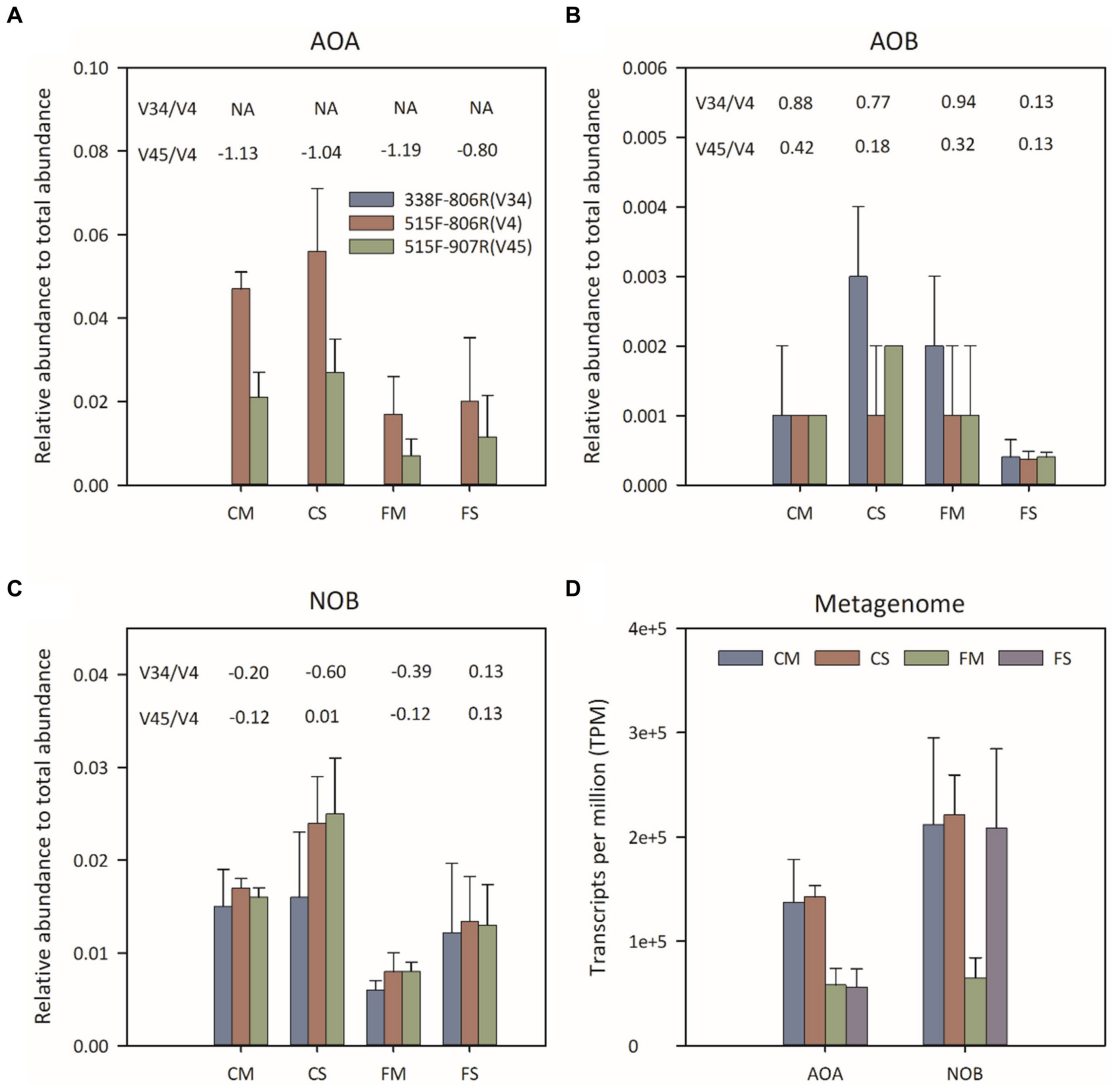
Relative abundance of Thaumarchaeota **(A)**, AOB **(B)** and NOB **(C)** 16S rRNA genes by amplicon sequencing and transcripts per million (TPM) values of predicted AOA and NOB **(D)** 16S rRNA by metagenomic sequencing. The number above the histogram indicated the log2-transformed fold change in the relative abundances by using 338F-806R(V34) or 515F-907R(V45) compared to using 515F-806R(V4), respectively. A positive value indicated significantly higher relative abundance by using 338F-806R(V34) or 515F-907R(V45) than 515F-806R(V4); a negative value indicated significantly lower relative abundance by using 338F-806R(V34) or 515F-907R(V45) than 515F-806R(V4). The error bars represent the standard deviation for each treatment.

All 16S rRNA sequences that could be assigned to the AOA, AOB and NOB of phyla Thaumarchaeota, Nitrosospira and Nitrospira, respectively, were chosen to assess the distribution of soil nitrifiers (Figures 2A–C). The Thaumarchaeota 16S rRNA sequence was rarely detected using 338F-806R. The average relative abundance of Thaumarchaeota detected by using 515F-806R (ranging from 1.66 to 5.59%) was higher than that detected by using 515F-907R (ranging from 0.73 to 2.72%) (Figure 2A). The distribution patterns of the relative abundance of Thaumarchaeota among different treatments were similar when 515F-806R and 515F-907R were used. The relative abundance of Thaumarchaeota was greater under controlled irrigation treatment than under flooding irrigation treatment. The relative abundance of AOB was significantly lower than that of Thaumarchaeota and ranged from 0.04 to 0.25%. AOB showed consistently higher proportions in the 338F-806R and 515F-907R data, than in 515F-806R data. Different primers pairs showed significant change in the relative proportion of NOB. For example, primers 338F-806R and 515F-907R resulted in higher proportions of NOB in FS treatment and lower proportions of NOB in CS and FM treatments, compared to results from 515F-806R.

### Comparison of community structure of nitrifiers from 16S rRNA gene amplicon sequences

**Figure 3:**
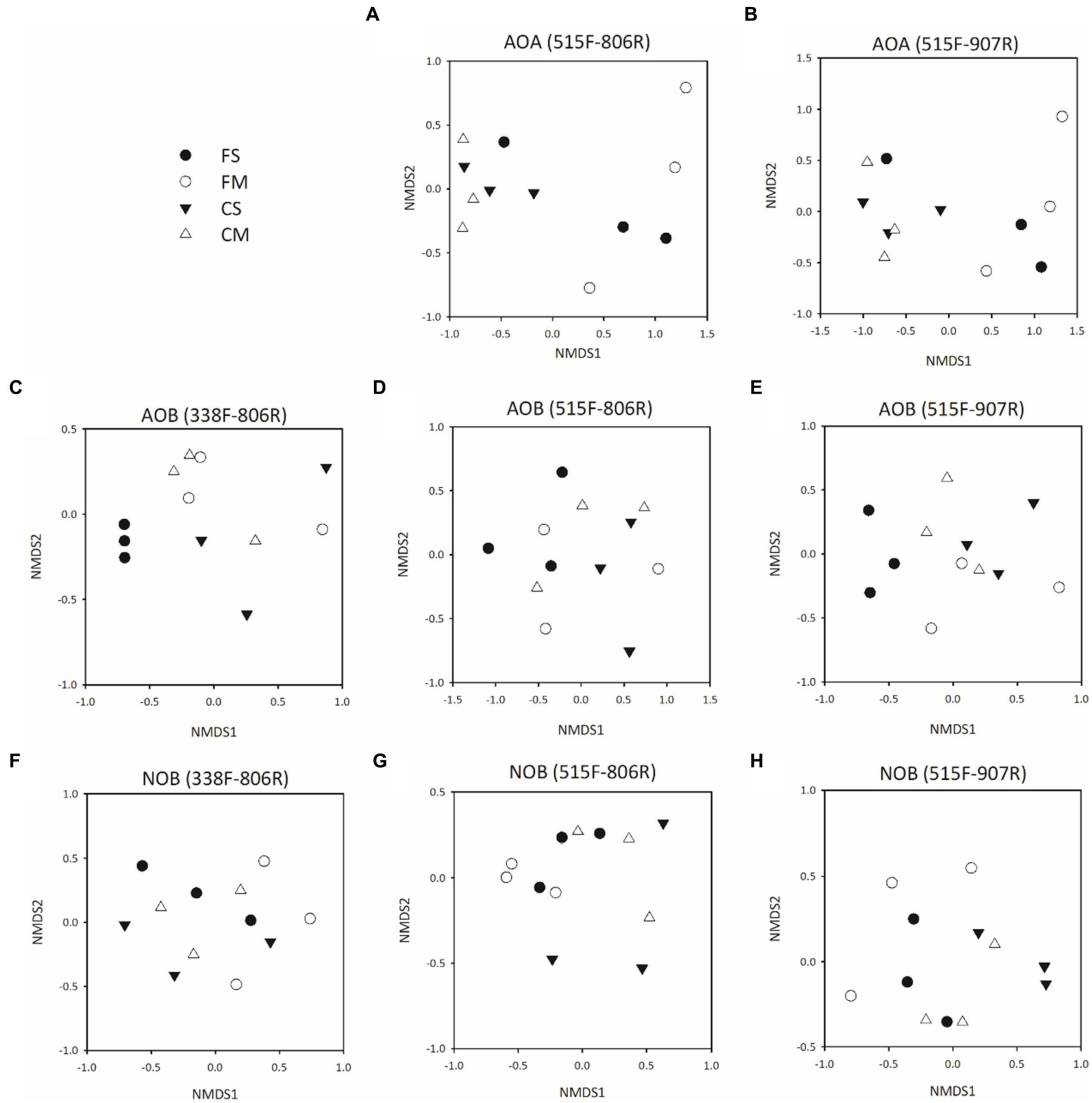
Microbial community structure of Thaumarchaeota **(A,B)**, AOB **(C–E)** and NOB **(F–H)** 16S rRNA genes in soils with different treatments by using different primer pairs as described by NMDS plots. All designations are the same as those in Figure 1.

The community composition of Thaumarchaeota was significantly different between controlled irrigation and flooding irrigation treatments for both 515F-806R and 515F-907R primers (*p* < 0.05) (Figures 3A,B; Supplementary Table S5). However, no significant difference was detected in the Thaumarchaeota community between organic fertilizer and straw amendment treatments when 515F-806R or 515F-907R primers were used (*p* > 0.05). For AOB, no significant difference was observed in community structure between different irrigation treatments or fertilizer amendments (Figures 3C–E; Supplementary Table S5). A difference was only detected in the NOB community structure between different irrigation treatments by use of 515F-806R and 515F-907R (*p* < 0.05) (Figures 3F–H; Supplementary Table S5).

### Comparison of phylotype distribution of nitrifiers from 16S rRNA gene amplicon sequences

**Figure 4:**
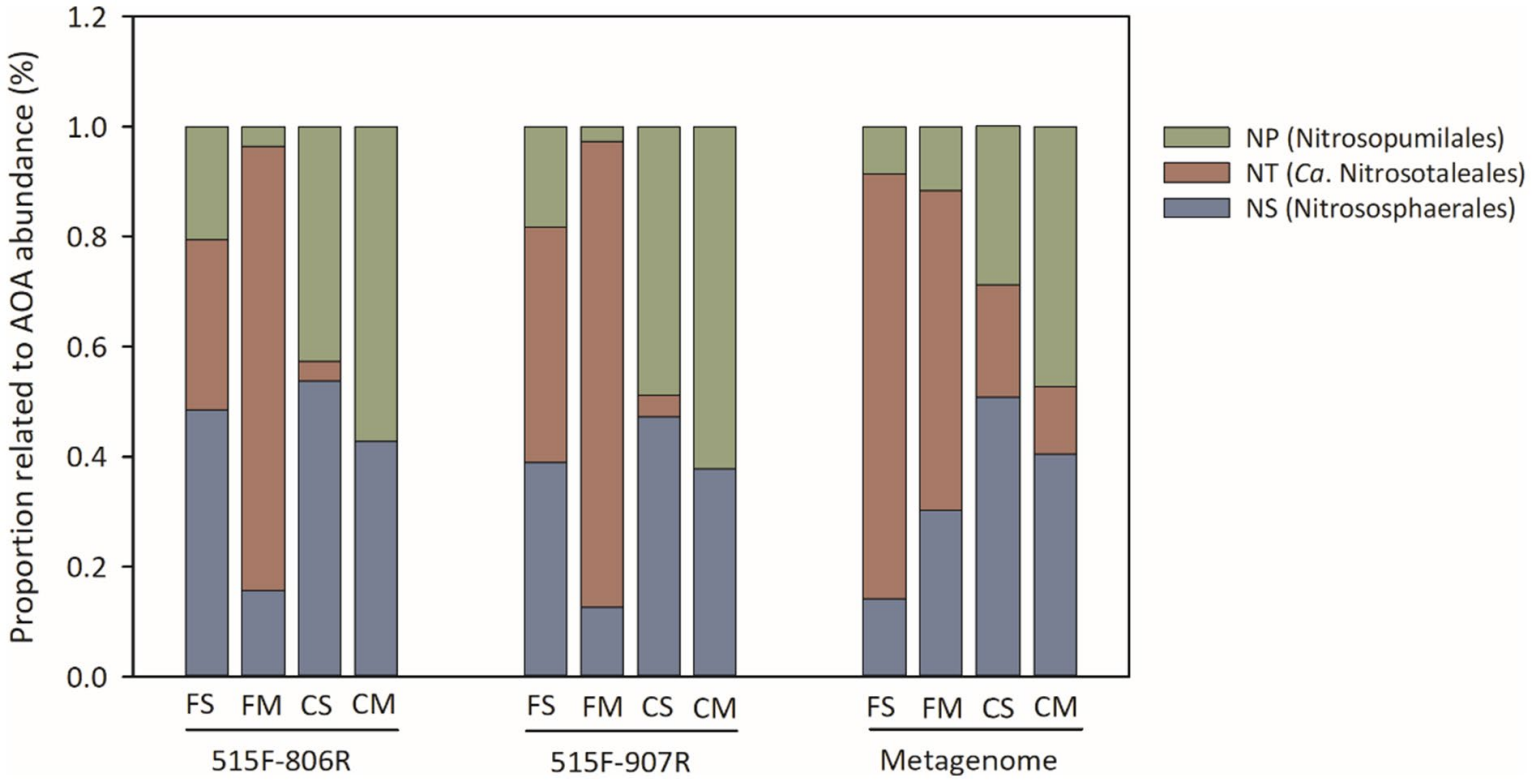
The relative abundance of different phylogenetic clades of AOA, estimated by amplicon sequencing of 16S rRNA genes and by metagenomic sequencing. All designations are the same as those in Figure 1.

A NJ phylogenetic tree of the 448 ASVs of Thaumarchaeota was constructed using the maximum composite likelihood method. The ASVs of Thaumarchaeota were branched into Nitrososphaerales (NS), *Ca.* Nitrosotaleales (NT) and Nitrosopumilales (NP) clades (Figure 4). The NT and NS clades of AOA were dominant Thaumarchaeota phylotypes under flooding irrigation treatment, while the NP and NS clades of Thaumarchaeota dominated under controlled irrigation treatment. The proportions of NT and NS clades ranged from 21.5–93.1% and 6.5–49.4%, respectively, under flooding irrigation treatment, whereas those of NP and NS clades ranged from 32.3–85.1% and 14.9–67.7%, respectively, under controlled irrigation treatment. All the ASVs of AOB belonged to Nitrosospira cluster 3. For NOB, Nitrospira lineage II was the dominant NOB phylotype in all the samples, ranging from 81.4 to 100%. Lineage I NOB was relatively higher under flooding irrigation treatment than under controlled irrigation treatment.

### Comparison of TPM value and phylotype distribution of nitrifiers from shotgun metagenomes

Twelve shotgun metagenomes were obtained with about 30 Gb per sample. The relative abundance of 16S rRNA taxons was evaluated with TPM value (Figure 2D). The relative abundance of the AOA 16S rRNA gene was higher under controlled irrigation treatment (0.10–0.20) than under flooding irrigation treatment (0.03–0.08). Moreover, no significant difference was observed between different amendments under the same irrigation treatment (*p* > 0.05). The phylotype distribution pattern of AOA was similar to the result of 16S rRNA sequencing. That is, the NT and NS clades of AOA were dominant AOA phylotypes under flooding irrigation treatment, while the NP and NS clades of AOA were dominant under controlled irrigation treatment. The relative abundance of the NOB 16S rRNA gene was lowest under FM treatment, while no significant difference was detected between FS, CS and CM treatments. No AOB 16S rRNA gene was detected from metagenomic data, which is consistent with the lower relative abundance of AOB from 16S rRNA amplicon sequencing.

## Discussion

The selection of the validated primer pairs for 16S rRNA hypervariable regions is critical in ensuring the characterization accuracy of microbial communities. In this study, 338F-806R lowered bacterial alpha diversity compared to 515F-806R and 515F-907R. This result concurs with a previous study (Birtel et al., 2015), where primer sets targeting the V4 region appeared to provide greater richness than those targeting the V34 and V45 regions. The V4 region has been recognized as a possible primer set for assessing bacterial diversity (Youssef et al., 2009; Klindworth et al., 2013; Zhang et al., 2018). The primer set 515F-806R targeting the V4 region is recommended by the EMP (Apprill et al., 2015; Parada et al., 2016). However, the primer sets targeting the V3-V4 region have shown good performance when applied to a plant-associated and soil bacterial microbiome (Thijs et al., 2017). Hence, the performance of primers may also depend on the source of samples.

Short-read sequencing (e.g., Illumina) technology poses a challenge when sequencing specific microbial guilds based on functional genes exceeding 500 bp. This suggests that evaluating whether universal 16S rRNA gene amplicons could generate information on such guilds. Data from this study demonstrated that 515F-806R and 515F-907R offered better performance in the establishment of AOA communities compared to 338F-806R. The use of 515F-806R and 515F-907R contributed to a much higher relative abundance of AOA than that of 338F-806R. This finding indicated the limitations of 338F-806R in measuring AOA communities. Shotgun metagenome-derived 16S rRNA gene data that could be more precise than amplicon-based sequencing approaches in characterizing microbial profiles (Brooks et al., 2015). In this study, the relative abundance values of AOA calculated by using 515F-806R and 515F-907R were lower compared to those derived from metagenomic data. This seems to be partially attributable to the loss of some sequences caused by metagenomic assembly or polymerase chain reaction (PCR) amplification bias. Furthermore, the distribution patterns of relative abundance of AOA were similar among different treatments, which indicated that the irrigation method was the key factor influencing the community structure of AOA. Moreover, the phylotype distribution of AOA was consistent with metagenomic and PCR amplification sequencing when 515F-806R and 515F-907R were used. This suggests that the NT and NS clades of AOA were dominant under flooding irrigation treatment, while the NP and NS clades of AOA were dominant under controlled irrigation treatment. Collectively, these results indicated that 515F-806R and 515F-907R offered similar performance in assessing the relative abundance and community structure of AOA.

AOA numerically dominated AOB in all samples, which suggested that AOA might dominate the process of ammonia oxidation in the soils sampled. This phenomenon has two possible explanations. First, organic fertilizer and straw amendment stimulate the growth of AOA. Previous studies reported that the abundance of AOA increased with the addition of organic N compounds, but did not respond to the addition of ${\mathrm{NH}}_{4}^{+}$ (Levičnik-Höfferle et al., 2012; Zhou et al., 2015; Zhongjun et al., 2020). Pure cultures of AOA grew after the addition of organic compounds to the growth medium (Tourna et al., 2011; Lehtovirta-Morley et al., 2014), while AOB thrived in environments with higher concentrations of inorganic NH_4_^+^. Second, AOA prefers to grow under irrigated conditions compared to AOB. Oxygen availability influenced by irrigation method has been identified as an important environmental factor that leads to the niche specialization and evolution of Thaumarchaeota (Ren et al., 2019). Available environmental and culture-based data reveal that AOA has a higher affinity for O_2_ than AOB (Martens-Habbena et al., 2009; Park et al., 2010; Bristow et al., 2016; Qin et al., 2017).

The findings of this study demonstrate that the differentiation in irrigation methods was related to the phylotype distribution of AOA, that NT and NS were dominant AOA clades under flooding irrigation treatment, while the NP and NS clades of AOA dominated in the soils with controlled irrigation treatment. The results of this study concur with previous findings that most AOA clades in soils and sediments belong to the NS lineage (Alves et al., 2018). The NP clade was mostly comprised of marine AOA (Qin et al., 2016), with the highest abundance often detected in deep ocean sediments and oxygen minimum zones (Bristow et al., 2016; Ren et al., 2019; Reji et al., 2022). The NT clade of AOA was also abundant in deep lakes (Ren et al., 2019). The high affinity for oxygen of members of NP could explain the fact that they were numerically predominant under controlled irrigation treatment. However, NT rather than NP dominated under flooding irrigation treatment. NT is an acidophilic lineage typically found in acidic soils or at least with pH < 7.5 (Gubry-Rangin et al., 2011; Prosser and Nicol, 2016). This might explain the dominance of the NT clade under flooding irrigation treatment, where the soil pH was relatively low. Further research using genomic tools remains to be done to reveal the metabolic properties and ecological adaptation of these AOA lineages under different irrigation treatments.

The findings of this study reveal various performances of NOB in terms of relative abundance using different primer pairs. *Nitrospira* sublineage II showed a strong dominance of the NOB community in all samples, consistent with the vast phylogenetic diversity and environmental distribution of *Nitrospira* sublineage II (Daims et al., 2001; Daims and Wagner, 2018; Vijayan et al., 2021). With the potential to fully oxidize ${\mathrm{NH}}_{4}^{+}$ to ${\mathrm{NO}}_{3}^{-}$ , the recently discovered comammox bacteria belong to *Nitrospira* lineage II (Daims et al., 2015; Van Kessel et al., 2015). However, comammox *Nitrospira* does not form a monophyletic clade within *Nitrospira* lineage II based on the 16S rRNA gene. Future studies should determine the activity of comammox under irrigation treatment.

## Conclusion

Taken together, this study evaluated the performance of primer pairs 338F-806R, 515F-806R, and 515F-907R in characterizing bacterial and nitrifiers’ communities, in comparison to shotgun metagenome-based results. Overall, 515F-806R and 515F-907R offered better performance than 338F-806R for the overall bacterial community and AOA, whereas the performance of these three primer sets were various for AOB and NOB communities. Despite the differentiation of bacterial and nitrifiers’ community composition among each primer set, the results of nitrifiers’ phylotype distribution were similar in this study. The AOA numerically dominated over AOB, and the irrigation method significantly influenced AOA community structure. Our present findings suggested the potential activity of AOA in nitrification, and the NP and NT clades of the AOA in particular, might have distinct environmental adaptation strategies. However, microbial abundance does not necessarily mirror activity. Future research could employ a DNA-SIP technique, which provides a powerful means to establish a direct link between biogeochemical processes and the taxonomic identities of active microorganisms involved in the processes (Jia et al., 2019). Furthermore, genomic tools could be used to clarify the ecological adaptation of these AOA lineages under different irrigation treatments.

## Data availability statement

The original contributions presented in the study are publicly available. The names of the repository/repositories and accession number(s) can be found in the article/Supplementary material.

## Author contributions

XZ: Writing – review & editing, Writing – original draft, Methodology, Investigation, Data curation. XL: Writing – review & editing, Writing – original draft, Methodology, Funding acquisition. ML: Writing – original draft, Investigation. WL: Writing – original draft, Methodology, Investigation. JX: Writing – review & editing, Writing – original draft, Supervision. YL: Writing – review & editing, Writing – original draft, Methodology, Investigation, Funding acquisition, Data curation.

## Footnotes

^1^ https://github.com/tseemann/barrnap
