# Supplementary material for: Comparative evaluation of 16S rRNA primer pairs in identifying nitrifying guilds in soils under long-term organic fertilization and water management
Source: Front Microbiol. 2024 Jul 15;15:1424795. doi: 10.3389/fmicb.2024.1424795 (PMC11284604; doi:10.3389/fmicb.2024.1424795)
Supplement: Supplementary file 1 [file Data_Sheet_1.pdf]

Supplementary material for:

Comparative evaluation of 16S rRNA primer pairs in identifying nitrifying guilds in soils under long-term organic fertilization and water management

Xue Zhou, Xiaoyin Liu, Meiyu Liu, Weixuan Liu, Junzeng Xu, Yawei Li*

*** Corresponding author**

Dr Yawei Li

College of Agricultural Science and Engineering

Hohai University

Nanjing, 211100

China

E-mail: yaweizx@hhu.edu.cn

Table S1 Physiochemical properties of soil samples.

Treatment*	SWC	pH	NH_4^+-N (mg/kg)	NO_3^--N (mg/kg)	TC (g/kg)	TN (g/kg)	C/N
FS	0.32 ± 0.02 a	5.83 ± 0.12 c	0.38 ± 0.09 a	0.80 ± 0.04 a	16.24 ± 2.68 c	1.75 ± 0.27 a	9.29 ± 0.32 b
FM	0.32 ± 0.03 a	6.49 ± 0.39 b	0.34 ± 0.03 a	0.65 ± 0.16 b	21.41 ± 1.82 a	1.58 ± 0.37 b	14.31 ± 2.96 a
CS	0.26 ± 0.06 b	6.89 ± 0.37 a	0.38 ± 0.03 a	0.54 ± 0.13 b	16.31 ± 2.51 c	1.67 ± 0.26 a	9.78 ± 0.15 b
CM	0.24 ± 0.04 b	6.89 ± 0.40 a	0.39 ± 0.05 a	0.68 ± 0.14 b	19.55 ± 2.37 b	1.48 ± 0.34 b	13.53 ± 2.77 a

***** Sample treatments include FS (flooding irrigation and straw returning management), FM (flooding irrigation and organic fertilizer management), CS (controlled irrigation and straw returning management) and CM (controlled irrigation and organic fertilizer management). Different letters above indicate statistically significant differences (P<0.05) by ANOVA analysis.

Table S2 Primers and conditions used in this study.

Primer Name	Primer sequence (5'-3')	Target V region	Thermal Profile
338F	ACTCCTACGGGAGGCAGCA	V3-V4	94°C, 5min; 32×(94°C, 30s; 54°C, 30s; 72°C, 45s); 72℃,10min; hold at 4℃
806R	GGACTACHVGGGTWTCTAAT		
515F	GTGYCAGCMGCCGCGGTAA	V4	
806R	GGACTACNVGGGTWTCTAAT		
515F	GTGCCAGCMGCCGCGG	V4-V5	
907R	CCGTCAATTCMTTTRAGTTT		

Table S3 Accession numbers of 16S rRNA gene and metagenomic sequences.

Treatment	16S rRNA gene			Metagenome
	338F-806R	515F-806R	515F-907R	
FS-1	SAMN36977440	SAMN36977452	SAMN36977464	SAMN36988134
FS-2	SAMN36977446	SAMN36977458	SAMN36977470	SAMN36988135
FS-3	SAMN36977448	SAMN36977460	SAMN36977472	SAMN36988136
FM-1	SAMN36977442	SAMN36977454	SAMN36977466	SAMN36988137
FM-2	SAMN36977444	SAMN36977456	SAMN36977468	SAMN36988138
FM-3	SAMN36977450	SAMN36977462	SAMN36977474	SAMN36988139
CS-1	SAMN36977441	SAMN36977453	SAMN36977465	SAMN36988140
CS-2	SAMN36977447	SAMN36977459	SAMN36977471	SAMN36988141
CS-3	SAMN36977449	SAMN36977461	SAMN36977473	SAMN36988142
CM-1	SAMN36977443	SAMN36977455	SAMN36977467	SAMN36988143
CM-2	SAMN36977445	SAMN36977457	SAMN36977469	SAMN36988144
CM-3	SAMN36977451	SAMN36977463	SAMN36977475	SAMN36988145

Table S4 *P* values of ANOSIM of bacterial community structure among different treatments by using different primer pairs. The designation FS and FM indicate flooding irrigation and straw returning management and flooding irrigation and organic fertilizer management, respectively. The designations CS and CM indicate controlled irrigation and straw returning management and controlled irrigation and organic fertilizer management, respectively. *$P < 0.05$

	FS-CS	FS-FM	FS-CM	CS-FM	CS-CM	FM-CM
338F-806R	0.1	0.1	0.1	0.1	0.5	0.1
515F-806R	0.1	0.1	0.1	0.1	0.4	0.1
515F-907R	0.1	0.1	0.1	0.1	0.3	0.1

Table S5 *P* values of ANOSIM of AOA, AOB and NOB community structure among different irrigation and fertilizer treatments. ‘S-M’ represent the comparison between straw and organic matter. ‘F-C’ represent the comparison between flooding and controlled irrigation. *$P < 0.05$.

Nitrifier	Primer	S-M	F-C
AOA	338F-806R	-	-
	515F-806R	0.821	0.006*
	515F-907R	0.865	0.004*
AOB	338F-806R	0.703	0.336
	515F-806R	0.710	0.183
	515F-907R	0.484	0.052
NOB	338F-806R	0.096	0.205
	515F-806R	0.502	0.014*
	515F-907R	0.572	0.021*
